# Microfluidic Coupling of Step Emulsification and Deterministic Lateral Displacement for Producing Satellite-Free Droplets and Particles

**DOI:** 10.3390/mi14030622

**Published:** 2023-03-08

**Authors:** Guangchong Ji, Yusuke Kanno, Takasi Nisisako

**Affiliations:** 1 Department of Mechanical Engineering, School of Engineering, Tokyo Institute of Technology, Tokyo 152-8550, Japan; 2 Institute of Innovative Research, Tokyo Institute of Technology, Yokohama 226-8503, Japan

**Keywords:** step emulsification, deterministic lateral displacement, droplet separation, satellite droplets, polymeric microspheres

## Abstract

Step emulsification, which uses a geometry-dependent mechanism for generating uniformly sized droplets, has recently gained considerable attention because of its robustness against flow fluctuations. However, like shear-based droplet generation, step emulsification is susceptible to impurities caused by satellite droplets. Herein, we demonstrate the integration of deterministic lateral displacement (DLD) to separate the main and satellite droplets produced during step emulsification. Step-emulsification nozzles (16 μm deep) in the upstream region of the proposed device were arrayed on the sidewalls of the main channel (91 μm deep). In the downstream region, the DLD micropillars were arrayed periodically with a critical diameter (cut-off value for size-based separation) of 37 μm. When an acrylate monomer and aqueous polyvinyl alcohol solution were infused as the dispersed and continuous phases, respectively, the nozzles produced monodisperse main droplets in the dripping regime, with an average diameter of ~60 μm, coefficient of variation (CV) value below 3%, and satellite droplets of ~3 μm. Upon entering the DLD region near the sidewall, these main and satellite droplets were gradually separated through the pillars based on their sizes. Finally, off-chip photopolymerization yielded monodisperse polymeric microspheres with an average diameter of 55 μm and a CV value of 2.9% (*n* = 202).

## 1. Introduction

Over the last decade, microfluidic droplet generation has been extensively investigated for numerous applications. Droplet microfluidics has exhibited considerable potential in biomedical applications, including single-cell analysis, 3D cell culture, and spheroid encapsulation [1,2,3,4]. In particular, microfluidic droplet-based digital polymerase chain reaction has been increasingly used in the industry [5]. In food science, microfluidic encapsulation of sensitive components for probiotic and nutrient delivery has been investigated [6]. Moreover, various functional microparticles have been synthesized from precursor droplets produced by microfluidic droplet generators [7,8,9].

Among the various microfluidic droplet generation processes, step emulsification, which uses a nozzle leading to a step facing a deeper main channel, has gained considerable attention [10,11]. In step emulsification, the break-off of a droplet is driven by interfacial tension, which primarily depends on the geometry and surface wetting of the nozzle. Therefore, unlike shear-based droplet generation [1,2,3,4,5,6,7,8,9], the size of the droplets in step emulsification is insensitive to the flow rates of the dispersed and continuous phases, and it can be easily predetermined by the nozzle geometry (typically the nozzle height). This robustness against flow fluctuation is also advantageous in massively parallelized nozzles for scaled-up production. For example, a millipede-shaped polydimethylsiloxane (PDMS) step-emulsification device with 550 parallelized triangular nozzles has been reported to generate monodisperse water-in-oil (W/O) droplets at a throughput of 150 mL/h [12]. Similarly, functional microparticles [13], complex double emulsions [14,15], and Janus droplets [16] have been produced via step emulsification. Meanwhile, as in shear-induced droplet generation, the pinched neck immediately before droplet break-off in step emulsification is also governed by the Plateau–Rayleigh instability [17]. Although this instability can produce satellite droplets in step emulsification, it has rarely been discussed in the literature thus far.

Several attempts have been made to develop shear-induced droplet generators for a continuous separation of the main and satellite droplets. For example, simple bifurcation geometries have been installed after T-shaped [18,19] or flow-focusing [20] droplet generators for sorting the main and satellite droplets. However, the main and satellite droplets must continue to flow on separate streamlines leading to different collection channels/outlets in these devices. Additionally, separating them is difficult if they are mixed in a confined channel. Meanwhile, deterministic lateral displacement (DLD) [21,22,23,24] micropillars, which enable continuous and size-based particle separation at high resolution, have also been coupled with a shear-based droplet generation for sorting the main and satellite droplets. Tottori et al. first used a device comprising a flow-focusing droplet generator and single DLD region to separate ~61 μm main droplets and 1–30 μm satellite droplets [25]. Subsequently, they demonstrated the fractionation of satellite droplets of various sizes using a device with multiple DLD regions. In a separate study, parallelizing eight devices on a single chip was reported to increase the throughput [26]. However, coupling with DLD for satellite-free step emulsification has not been reported thus far.

Herein, we report the coupling of step emulsification and DLD separation for producing monodisperse droplets and particles free of satellite droplet impurities. The microfluidic device has an upstream region where step-emulsification nozzles are arrayed on the sidewalls of the main channel, and the downstream region is filled with a periodic array of DLD micropillars. First, we describe how satellite droplets are generated when the main droplets break off at the exits of the nozzles. Next, we present how the main and satellite droplets migrate separately through the DLD micropillars based on their sizes. Finally, we describe how the separately collected main droplets are photopolymerized to produce monodisperse polymer microspheres.

## 2. Materials and Methods

### 2.1. Device Design and Mechanism

**Figure 1:**
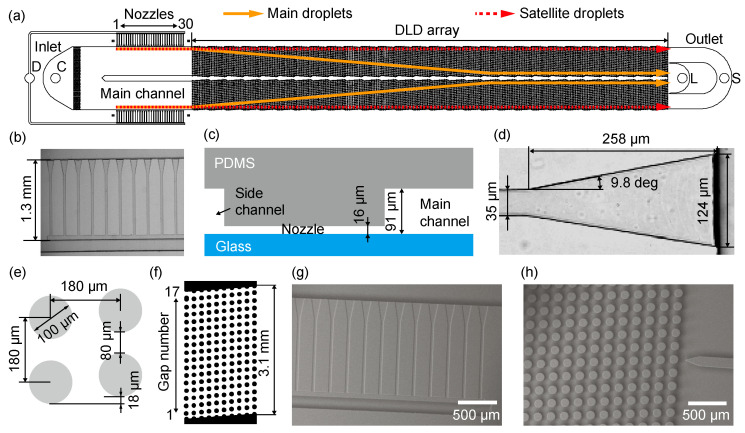
A microfluidic device comprising step-emulsification nozzles and deterministic lateral displacement (DLD) pillars for separating main and satellite droplets. (**a**) Schematic illustration of the top view of the device. (**b**) A photomicrograph of the nozzles. (**c**) A schematic cross-section of a nozzle connecting the side and main channels. (**d**) A photomicrograph of a triangular end of a nozzle. (**e**) A rhombic unit of the DLD pillars. (**f**) A single region containing the 10-column DLD pillars. (**g**,**h**) SEM images of the (**g**) nozzles and (**h**) pillars.

We designed a device comprising step emulsification nozzles and DLD micropillar arrays, as shown in Figure 1a. This device has a symmetric layout on both sides of the central wall. Two inlets for the dispersed and continuous phases are located in the upstream region of the device. Subsequently, 30 step-emulsification nozzles are arranged in two arrays (i.e., 60 nozzles in total) perpendicular to the main channel. A periodic array of DLD micropillars in the downstream region is arranged in the main channel, which leads to two outlets for collecting the main and satellite droplets separately. A device without pillars was also prepared for comparison (see Supplementary Materials, Figure S1).

The step-emulsification nozzles, with a length and depth of 1.3 mm and 16 μm, respectively, are arranged in parallel with a pitch of 240 μm, connecting the side and main channels with a depth of 91 μm (Figure 1b,c). Each nozzle has a triangular end opening toward the main channel. The nozzle width increases from 35 to 124 μm along a length of 258 μm at an angle of 9.8° (Figure 1d).

The region corresponding to the periodically arrayed DLD micropillars begins immediately after the main channel with arrayed nozzles. This DLD array consists of a rhombic unit cell with a pillar diameter of 100 μm and a gap of 80 μm between the pillars, and thus a pitch of 180 μm between two adjacent pillars (Figure 1e). The shift between two adjacent pillar columns is 18 μm, and 10 columns form a single DLD region with 17 row gaps (Figure 1f). This single DLD region repeats 28 times toward the outlets displacing the main droplets through the pillars toward the central wall.

The critical diameter *D*_c_, which is known as the cut-off value for size-based separation of particles in DLD, was approximated using the following empirical formula [27]:

*D*_c_ = 1.4 × *G* × (Δ*λ*/*λ*)^0.48^ (1)

where *G* is the gap between the pillars (80 μm), *λ* is the pitch between the pillars (180 μm), and Δ*λ* (18 μm) is the shift between the pillars. Thus, the *D*_c_ of our device was designed to be 37 μm.

The dispersed and continuous phases are supplied separately from the two inlets. The dispersed phase flows through the side channels (200 μm wide) and fills the arrayed nozzles. Monodisperse main droplets are formed at the end of the nozzles in the dripping regime, together with satellite droplets as the byproducts when the flow rates of the two phases are controlled properly. These droplets then follow the continuous phase flow in the main channel along the sidewalls to enter the DLD region downstream. The main droplets, whose diameters are larger than Dc, are expected to migrate laterally across the micropillar arrays at the shift angle in the displacement mode. In contrast, the satellite droplets are expected to follow a laminar flow with a general migration angle of zero in the zigzag mode (Figure 1a).

### 2.2. Device Fabrication

Microfluidic devices were fabricated using PDMS via standard soft lithography. A mold to replicate the step-emulsification nozzles (Figure 1g) and DLD pillars (Figure 1h) was fabricated in two steps. A region for the step-emulsification nozzles was first prepared. A silicon wafer (diameter 4 in; thickness 525 ± 25 μm; Canosis, Tokyo, Japan) was successively washed with acetone, ethanol, and pure water using an ultrasonic cleaner (B1510J-DTH, Bransonic Ultrasonics, CT, USA). A negative photoresist (SU-8 3025, Nippon Kayaku, Tokyo, Japan) was coated onto the silicon wafer using a spin coater (MS-B100, Mikasa, Tokyo, Japan) at 4500 rpm for 30 s and baked at 95 °C for 10 min on a hot plate (HI-1000, AS ONE, Osaka, Japan). Subsequently, the SU-8 layer was exposed to ultraviolet (UV) light at 200 mJ/cm^2^ through a laser-printed polyethylene terephthalate photomask (thickness 0.175 mm; resolution 25,400 dpi; Unno Giken, Tokyo, Japan) and first baked at 95 °C for 5 min, followed by baking at 150 °C for 20 min to obtain a 16 μm thick mold for the nozzles. To fabricate the mold structures for the other regions, including the DLD pillars, an additional layer of the photoresist (SU-8 3050, Nippon Kayaku, Tokyo, Japan) was coated on the same silicon wafer at 1200 rpm for 30 s, and it was baked at 95 °C for 45 min on a hot plate. After the same exposure, baking, and developing steps, a mold region with a height of 91 μm was obtained (Figure S2). Subsequently, the SU-8 mold was placed in a glass Petri dish, and its surface was modified to be hydrophobic by dripping 1 mL of chlorotrimethylsilane (Tokyo Chemical Industry, Tokyo, Japan) around it and sealing the dish for 5 min using aluminum foil. Subsequently, the SU-8 mold was baked at 110 °C for 10 min on a hot plate.

The PDMS precursor (Toray, Tokyo, Japan) was mixed with a curing agent (10:1 *w*/*w* ratio) and degassed in a desiccator (MVD-100; AS ONE, Osaka, Japan) for more than 30 min. The PDMS prepolymer mixture was then poured onto a SU-8 mold and baked on a hot plate at 80 °C for more than 2 h. After peeling off the solidified PDMS replica from the SU-8 mold, holes for inlets and outlets were produced using a punching tool (diameter of 1 mm; BPP-10F, Kai Industries, Gifu, Japan). The PDMS replica was sealed with a glass slide (76 mm × 26 mm; thickness 0.9–1.2 mm; Matsunami Glass, Osaka, Japan) using oxygen plasma treatment (flow rate of O_2_; 20 mL/min; 20 W, 1 Torr, 30 s; BP-1, Samco, Tokyo, Japan).

### 2.3. Surface Modification

After degassing for 15 min using the plasma treatment equipment, the PDMS device was treated with oxygen plasma (flow rate of O_2_; 20 mL/min; 100 W, 1 Torr, 3 min). Subsequently, an aqueous reagent containing a water-soluble polymer (0.5 mL; SPRA-202, Tokyo Ohka Kogyo, Kanagawa, Japan) was manually infused into the device using a disposable 1 mL syringe (Terumo, Tokyo, Japan). After wetting the microchannel surface for 1 min, it was washed with pure water and manually dried with air using a disposable syringe. The contact angles of pure water on the modified and unmodified PDMS surfaces were measured to be 3.2 ± 0.4° (*n* = 10) and 110.9 ± 2.8° (*n* = 10), respectively (Figure S3).

### 2.4. Chemicals

Acetone (>99%, FUJIFILM Wako Pure Chemical, Osaka, Japan), ethanol (>99.5%, FUJIFILM Wako Pure Chemical, Osaka, Japan), and pure water (Direct-Q, UV3, Merck, Hessen, Germany) were used to wash the silicon wafer and microspheres. A 2 wt% aqueous solution of polyvinyl alcohol (PVA; GL-03; *M*_w_ ~20,000 g/mol; 87–89% hydrolyzed; Mitsubishi Chemical, Tokyo, Japan) having a viscosity of 1.75 mPa s and a density of 1.00 g/cm^3^ was prepared for the continuous phase. A 1 wt% solution was prepared by adding 2-hydroxy-2-methylpropiophenone (Darocur 1173, BASF, Tokyo, Japan) to 1,6-hexanediol diacrylate (HDDA; Shin-Nakamura Kagaku, Tokyo, Japan) having a viscosity of 6.35 mPa s and a density of 1.02 g/cm^3^ for the photocurable dispersed phase.

### 2.5. Equipment

A gas-tight glass syringe (volume 1 mL; Hamilton, Nevada, USA) filled with the dispersed phase and a plastic syringe (volume 50 mL; Terumo, Tokyo, Japan) filled with the continuous phase, equipped with syringe needles (SNA-22G-C, Musashi Engineering, Tokyo, Japan) were linked to the inlets of the PDMS devices via polyethylene tubes (0.5 mm i.d., 1.0 mm o.d.; Hibiki #3, Kunii, Tokyo, Japan). These syringes were driven using syringe pumps (Legato 180 and KDS 210, KD Scientific, MA, USA). An inverted optical microscope (IX 73, Olympus, Tokyo, Japan) equipped with a high-speed video camera (Fastcam Mini AX50, Photron, Tokyo, Japan) was used to observe and record droplet formation and migration in the device (Figure S4). A free software, ImageJ (National Institutes of Health, MD, USA) was used to measure the droplets and particles. The contact angles of pure water on the coated and uncoated PDMS surfaces were measured using a drop shape analyzer (B100, Asumi Giken, Tokyo, Japan).

### 2.6. Preparation of Polymeric Microspheres

A UV light source (LA-410UV, Hayashi-repic, Tokyo, Japan) was used for the photopolymerization of the particles. After exposure for 30 s with an irradiation distance of around 15 cm, the polymerized particles were washed successively with pure water, acetone, and ethanol on a nylon mesh sheet (grid size: 42 μm × 42 μm; Tokyo Screen, Tokyo, Japan) sandwiched between a sampling flask (GSF-500, Toyo Roshi, Tokyo, Japan) and filter holder (GSA-01, Toyo Roshi, Tokyo, Japan). The dried microspheres were observed using a scanning electron microscope (FlexSEM 1000, Hitachi High-Tech, Tokyo, Japan) under high vacuum at an accelerating voltage of 10 kV.

## 3. Results and Discussion

### 3.1. Formation of Main and Satellite Droplets via Step Emulsification

**Figure 2:**
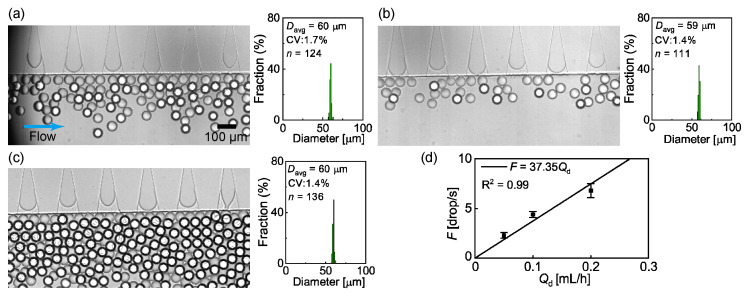
Step emulsification at different flow rates. (a–c) Formation of droplets and their size distributions when the disperse phase flow rate (*Q*_d_) was set at (**a**) 0.1, (**b**) 0.05, and (**c**) 0.2 mL/h while the continuous phase flow rate was kept at 10.0 mL/h. (**d**) Effect of *Q*_d_ on the droplet break-off frequency per nozzle (*F*).

Herein, we investigated how the step-emulsification nozzles could generate droplets using HDDA and an aqueous PVA solution as the dispersed and continuous phases, respectively. We observed the formation of similar-sized droplets in the dripping regime of all the 60 nozzles when the flow rates of the dispersed phase (*Q*_d_) and continuous phase (*Q*_c_) were 0.1 mL/h and 10.0 mL/h, respectively (Figure 2a, Video S1). At the triangular end of each nozzle, the curved HDDA/PVA interface repeatedly moved toward the nozzle edge, where the rapid growth of a droplet as well as rapid pinching of the neck was observed, which resulted in the final break-off of the main droplet. The main droplets were highly monodispersed, with an average diameter (*D*_avg_) of 60 μm and a coefficient of variation (CV) value of 1.7% (*n* = 124). The droplet production rate per nozzle (*F*) was 4.4 ± 0.3 drop/s (*n* = 60). The droplets did not coalesce around the nozzles.

Next, we varied *Q*_d_ for a constant *Q*_c_ (=10.0 mL/h) to investigate its effect on *D*_avg_ and *F*. No significant change was observed in the droplet size and size distribution, with *D*_avg_ = 59 μm and CV = 1.4% (*n* = 111), when *Q*_d_ decreased from 0.1 mL/h to 0.05 mL/h. However, *F* decreased to 2.3 ± 0.3 drop/s (*n* = 60), resulting in a less dense distribution of the droplets near the nozzles (Figure 2b). In contrast, when *Q*_d_ increased to 0.2 mL/h, similar-sized droplets were generated in the dripping regime, with *D*_avg_ = 60 μm and CV = 1.4% (*n* = 136), while *F* increased to 6.8 ± 0.7 drops/s (*n* = 60), resulting in a dense accumulation of the droplets (Figure 2c). The insensitivity of *D*_avg_ to *Q*_d_ and the linear relationship between *Q*_d_ and *F* (Figure 2d) were in good agreement with previously reported results [10,11].

When *Q*_d_ was further increased above 0.2 mL/h, the nozzles generated monodisperse droplets in the dripping regime. However, at a *Q*_d_ value above 0.2 mL/h, the densely packed droplets coalesced significantly before the DLD region (Figure S5a). This accumulation-induced coalescence was attributed to the presence of DLD pillars because such droplet coalescence was not observed under the same flow conditions in the device without pillars (Figure S5b). Meanwhile, given the droplet coalescence, the continuous-phase flow rate *Q*_c_ was also significant. For lower *Q*_c_ values, the droplets were densely packed at a lower *Q*_d_, resulting in coalescence before the DLD pillars (Figure S6a). The decreased droplet density, resulting from an increased *Q*_c_, resolved the issue of coalescence. A similar trend was also confirmed in the no-pillar device (Figure S6b). However, at a higher *Q*_c_, the number of activated nozzles working in the dripping regime decreased because of the increased pressure gradient along the main channel. In the subsequent experiments, the upper limits of *Q*_d_ and *Q*_c_ were set at 0.2 mL/h and 10.0 mL/h, respectively, because of the aforementioned limitations.

**Figure 3:**
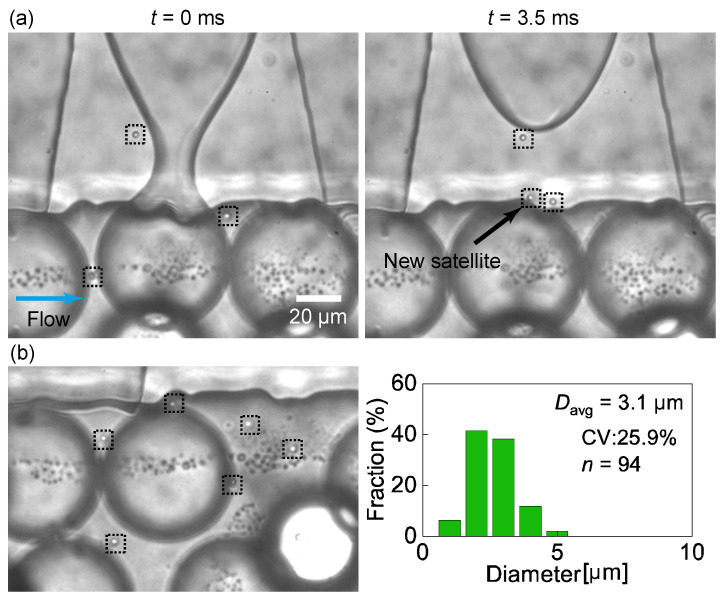
Formation of satellite droplets. (**a**) Snapshots of the satellite droplets (in the dashed squares) around a nozzle taken before (*t* = 0 ms) and after (*t* = 3.5 ms) the break-off of a main droplet. (**b**) Satellite droplets flowing around the nozzle edge and their size distribution. Flow rates were *Q*_d_ = 0.1 mL/h and *Q*_c_ = 10.0 mL/h.

In the magnified views, the formation of the satellite droplets is observed to be driven by the Plateau–Rayleigh instability (Figure 3a, Video S2). The high-speed video images confirm that satellite droplets are generated upon the break-off of the main droplet from the pinched neck. These satellite droplets exhibit a mean diameter of 3.1 μm, with a CV of 25.9% (*n* = 94, Figure 3b).

The satellite droplets were not carried away instantly by the continuous phase and continued to migrate in and out of the nozzles. This was presumably owing to the backflow of the continuous phase into the nozzles, which occurred repeatedly upon the rapid shrinking of the neck of the main droplet.

### 3.2. Separation of the Main and Satellite Droplets through DLD Pillars

**Figure 4:**
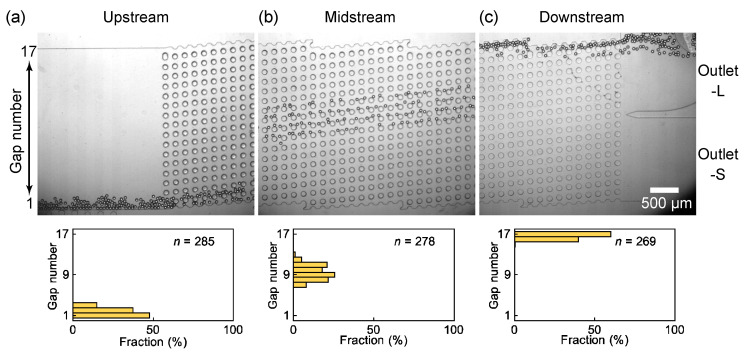
Spatial distribution of main droplets flowing through the DLD pillar arrays. (**a**) Main droplets entering the DLD region near the side wall. (**b**) Main droplets flowing through the midstream region in displacement mode. The droplets were counted in the 14th DLD section. (**c**) Fully displaced main droplets entering outlet-L. Flow rates were *Q*_d_ = 0.1 mL/h and *Q*_c_ = 10.0 mL/h.

The main and satellite droplets generated at the nozzles entered the DLD region and migrated separately through the pillars based on their sizes. Figure 4 shows the migration of the main droplets through the DLD pillars for the flow rates of *Q*_d_ = 0.1 mL/h and *Q*_c_ = 10.0 mL/h (Video S3). The Reynolds number under this flow condition was ~0.2, indicating laminar flow through the pillars. After their generation at nozzles, the main droplets with *D*_avg_ ~60 μm continue to flow near the sidewall with the nozzle array and enter the DLD region one by one through the gaps near the wall, each with a width of 80 μm (Figure 4a). Only slight droplet accumulation can be observed at the DLD entrance, and no deformation or coalescence of the droplets is observed. These main droplets begin to migrate toward the central wall in the displacement mode. In the midstream region, the main droplets flow in the displacement mode through the slightly increased number of gaps (gaps 7–13, Figure 4b) toward the central wall. This broadened distribution indicates that the main droplets were too dense to follow the DLD principle strictly in the initial sections. In the downstream region, the main droplets are sufficiently displaced, flowing through gaps 16–17 near the central wall, and are collected via outlet L with a 100% efficiency; however, no main droplets flow into outlet S (Figure 4c). Thus, the main droplets, with a diameter larger than *D*_c_ (37 μm), flow through the pillars in the displacement mode, as expected. During migration, some of the main droplets deviate from the expected paths, indicating partial displacement because of interactions between closely flowing droplets.

**Figure 5:**
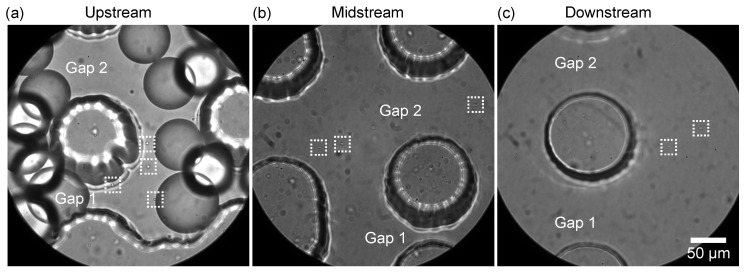
Satellite droplets flowing through the DLD pillars. (**a**–**c**) Satellite droplets (in the dashed white squares) flowing through the pillars in zigzag mode in the (**a**) upstream, (**b**) midstream, and (**c**) downstream regions. Flow rates were *Q*_d_ = 0.1 mL/h and *Q*_c_ = 10.0 mL/h.

Additionally, the flow of satellite droplets through the DLD region was investigated. In the magnified view, the satellite droplets can be observed to enter the DLD region near the sidewall (gaps 1–2) together with the main droplets (Figure 5a, Video S4). In the midstream region, unlike the main droplets, the satellite droplets maintain their position in the vertical direction with respect to the flow and move near the sidewall (gap 1–2, Figure 5b) in a zigzag mode. In the downstream region, the satellite droplets still flowing (gaps 1–2, Figure 5c) are collected from outlet S.

Thus, in our device with the DLD pillars, the satellite droplets, whose diameters (~3.1 μm) were smaller than *D*_c_ (37 μm), moved along a path different from that of the main droplets. This allowed a complete separation of the main and satellite droplets. Upon increasing *Q*_d_ to 0.2 mL/h while maintaining *Q*_c_ at 10.0 mL/h, a similar separation of the main and satellite droplets can be observed, despite an increased spatial density of the main droplets (Figures S7 and S8).

**Figure 6:**
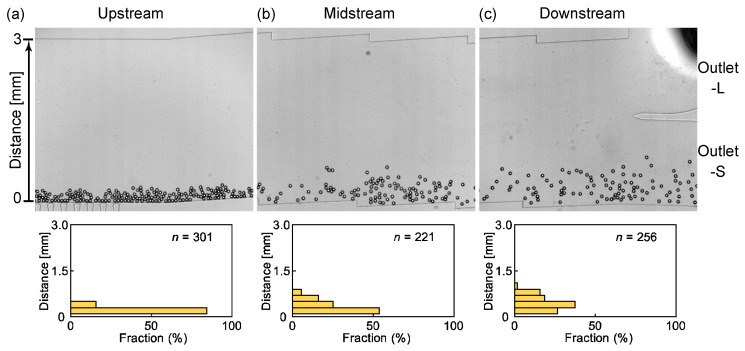
Spatial distribution of main droplets flowing in the no-pillar device. (**a**–**c**) Main droplets flowing through (**a**) the upstream region immediately after the nozzles, (**b**) midstream region, and (**c**) downstream region before the outlets. Flow rates were *Q*_d_ = 0.1 mL/h and *Q*_c_ = 10.0 mL/h.

**Figure 7:**
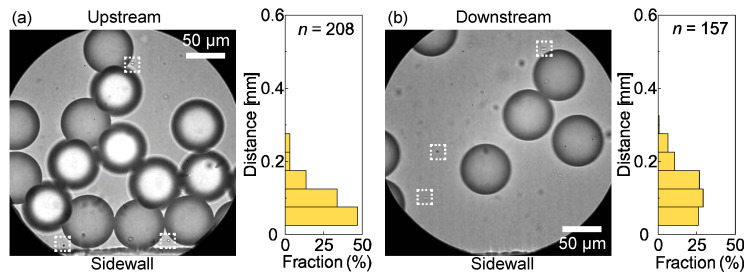
Satellite droplets flowing in the no-pillar device. (**a**) The satellite droplets (in the dashed white squares) flowing in the (**a**) upstream and (**b**) downstream regions and their distances from the sidewall. Flow rates were Q_d_ = 0.1 mL/h and Q_c_ = 10.0 mL/h.

For comparison, we also investigated the flow of droplets in a device without DLD pillars. After their generation at the nozzles, the main droplets continue to flow near the sidewall without any accumulation because no pillars prevent their motion (Figure 6a). During their migration in the midstream region, a slight displacement from the sidewall is observed for some droplets (Figure 6b). The localized and temporal flow disturbance generated by the droplet–sidewall interactions and the droplet–droplet interactions between closely flowing droplets might be the cause of this displacement. In the downstream region, the main droplets continue to flow near the sidewall at a distance of 0–1.0 mm (Figure 6c) and are collected at outlet S. In the magnified views of the upstream and downstream regions, the satellite droplets flowing close to the sidewall with a maximum distance of 0.3 mm and entering outlet S are observed (Figure 7). A similar droplet migration is observed when *Q*_d_ is increased to 0.2 mL/h (Figure S9). These results indicate that a device without DLD pillars cannot separate the main and satellite droplets.

### 3.3. Characterization of the Droplets and Particles

**Figure 8:**
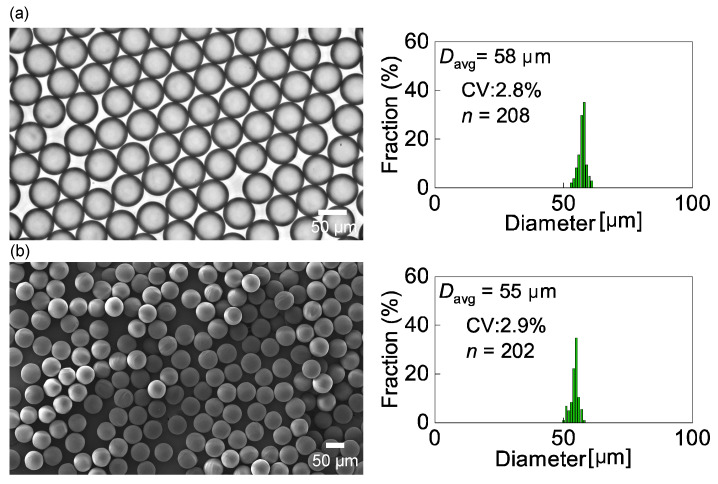
Collected main droplets and polymeric microspheres. (**a**) A photomicrograph of the main droplets collected from outlet-L and their size distribution. (**b**) An SEM image of the UV-polymerized particles and their size distribution.

The main droplets separated from the satellite droplets were collected in an off-chip Petri dish for size measurements. The collected main droplets were highly monodisperse, with a mean diameter of 58 μm and CV of 2.8% (*n* = 208, Figure 8a). The mean diameter (58 μm) of the collected droplets was slightly smaller than their diameter immediately after generation (60 μm) because the HDDA is slightly soluble in water (0.36 g/L at 20 °C). A portion of the HDDA droplets may have dissolved in the surrounding continuous phase during their migration in the device and collection in the Petri dish.

The main droplets were subsequently exposed to UV light for photopolymerization. As shown in Figure 8b, we obtained crosslinked polymeric microspheres with a mean diameter of 55 μm and a CV of 2.9%, which exhibited monodispersity similar to that of the precursor droplets. The shrinkage ratio of the polymer spheres from the precursor droplets upon photopolymerization was approximately 5.5%.

### 3.4. Advantages, Limitations, and Scope of the Device

The device proposed in this study has some advantages over previously reported droplet-sorting devices comprising shear-induced droplet makers and DLD pillars. Firstly, our device does not require an additional sheath fluid or flow-rate tuning to pre-focus the droplet stream in front of the DLD pillars because the droplets produced at the nozzles on the sidewall enter the DLD region near the sidewall. Therefore, our device has a significantly simpler design and operation than previously reported devices [25,26,28,29]. Secondly, unlike a sheathless device comprising a flow-focusing droplet maker and DLD pillars [25,26] which cannot utilize a half side of the DLD region, our device can utilize the entire DLD region efficiently. Thirdly, compared with the shear-induced systems, the number of step-emulsification nozzles in our device can be easily increased for scaled-up droplet processing with a smaller footprint [26]. Finally, step emulsification is known to be more robust to flow fluctuations than the shear-based droplet generation [10,11].

Our device can have broader applications. For example, satellite droplets of various sizes can be fractionated by arranging multiple DLD sections with various *D*_c_ values [25]. In addition to oil-in-water droplets, our device can process water-in-oil droplets if the channel surface is hydrophobic.

However, the accumulation and coalescence of droplets in front of the DLD pillars currently limit the monodispersity and/or productivity of our device. To overcome this limitation, we considered the following two promising approaches. The first involves maximizing the gap size between the pillars by setting *D*_c_ closer to the size of the main droplets, with a decreased shift between the pillar columns (Equation (1)). The second involves placing the DLD pillars in front of the nozzles to guide the main droplets away from the nozzles immediately following their formation. However, in this case, the pressure gradient would have to be carefully considered to avoid nozzle deactivation. Further investigations based on these approaches are currently underway.

## 4. Conclusions

We demonstrated the microfluidic production of monodisperse satellite-free droplets and particles by attaching DLD micropillar arrays to a cross-flowing step emulsifier. We first confirmed that the 60 nozzles arrayed upstream produced highly monodisperse main droplets (diameter ~60 μm; CV less than 2%) and satellite droplets (diameter ~3 μm). These main and satellite droplets were then separately collected with 100% purity using the downstream DLD micropillars with a *D*_c_ of 37 μm. Finally, monodisperse polymeric microspheres were obtained by photopolymerizing the main droplets free of satellite droplets. Our proposed device offers a promising protocol for conventional step emulsification to improve the purity of the product.

## Data Availability

The data presented in this study are available from the corresponding author on reasonable request.

## Supplementary Materials

The following supporting information can be downloaded at: https://www.mdpi.com/article/10.3390/mi14030622/s1, Figure S1: A step-emulsification device without DLD pillars, Figure S2: Scanning electron microscopy images of the SU-8 mold for PDMS casting, Figure S3: Contact angle measurements, Figure S4: Overall experimental setup, Figure S5: Droplet accumulation and coalescence in the devices, Figure S6: Effect of the flow conditions on the formation of droplets and their coalescence, Figure S7: Main droplet migration through the DLD pillars at *Q*_d_ = 0.2 mL/h and *Q*_c_ = 10.0 mL/h, Figure S8: Satellite droplet migration through the DLD pillars at *Q*_d_ = 0.2 mL/h and *Q*_c_ = 10.0 mL/h, Figure S9: Main droplet migration in the no-pillar device at *Q*_d_ = 0.2 mL/h and *Q*_c_ = 10.0 mL/h, Video S1: Step emulsification at a low magnification, Video S2: Step emulsification at a high magnification, Video S3: Main droplet migration through the DLD pillars, Video S4: Satellite droplet migration through the DLD pillars.
