# Supplementary material for: Microfluidic Coupling of Step Emulsification and Deterministic Lateral Displacement for Producing Satellite-Free Droplets and Particles
Source: Micromachines (Basel). 2023 Mar 8;14(3):622. doi: 10.3390/mi14030622 (PMC10055132; doi:10.3390/mi14030622)
Supplement: Supplementary file 1 [file micromachines-14-00622-s001.zip › micromachines-2266134-Supplementary Materials.pdf]

micromachines

Supplementary Material

Microfluidic Coupling of Step Emulsification and Deterministic Lateral Displacement for Producing Satellite-Free Droplets and Particles

Guangchong Ji, Yusuke Kanno and Takasi Nisisako*

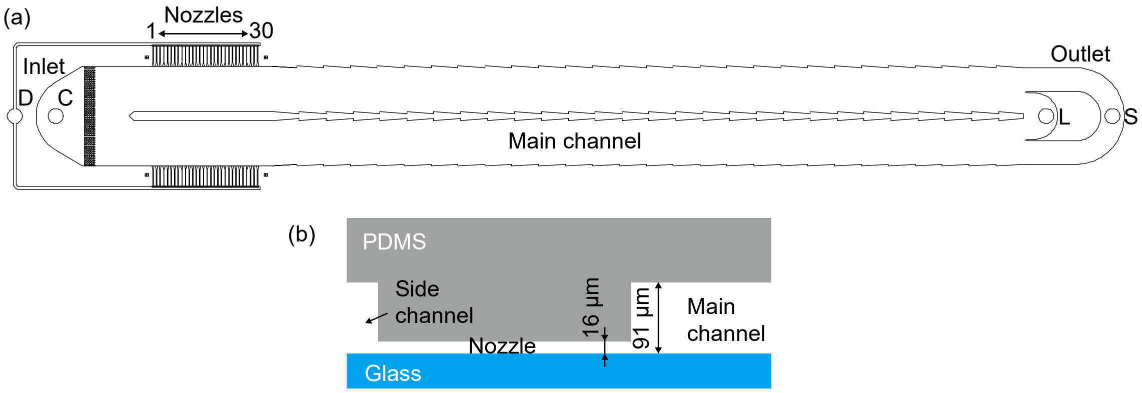

Figure S1. A step-emulsification device without DLD pillars. (**a**) Top view of the device. (**b**) Cross-sectional view of the upstream main channel with the nozzles.

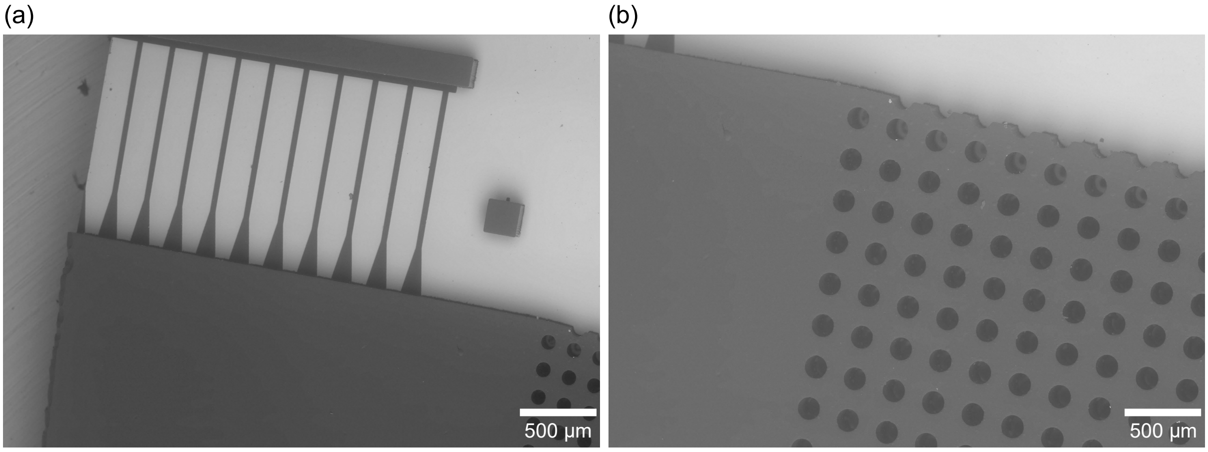

Figure S2. Scanning electron microscopy images of the SU-8 mold for PDMS casting. (**a**) A region for the step-emulsification nozzles. (**b**) A region for deterministic lateral displacement (DLD) micropillars.

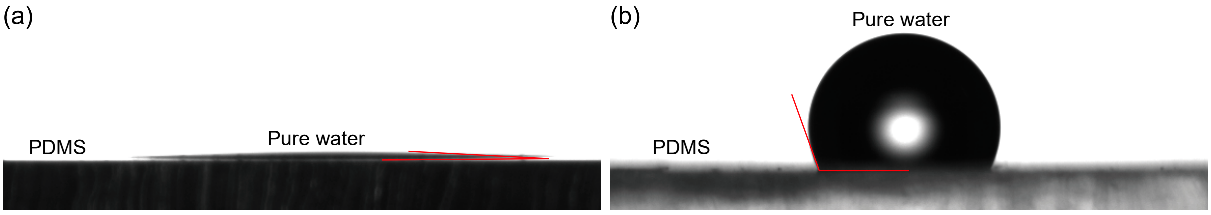

Figure S3. Measurement of contact angles of pure water on a PDMS chip with or without SPRA-202 coating. (**a**) A PDMS chip coated with SPRA-202. The measured contact angle was 3.2 ± 0.4° (n = 10). (**b**) Uncoated PDMS chip. The measured contact angle was 110.9 ± 2.8° (n = 10).

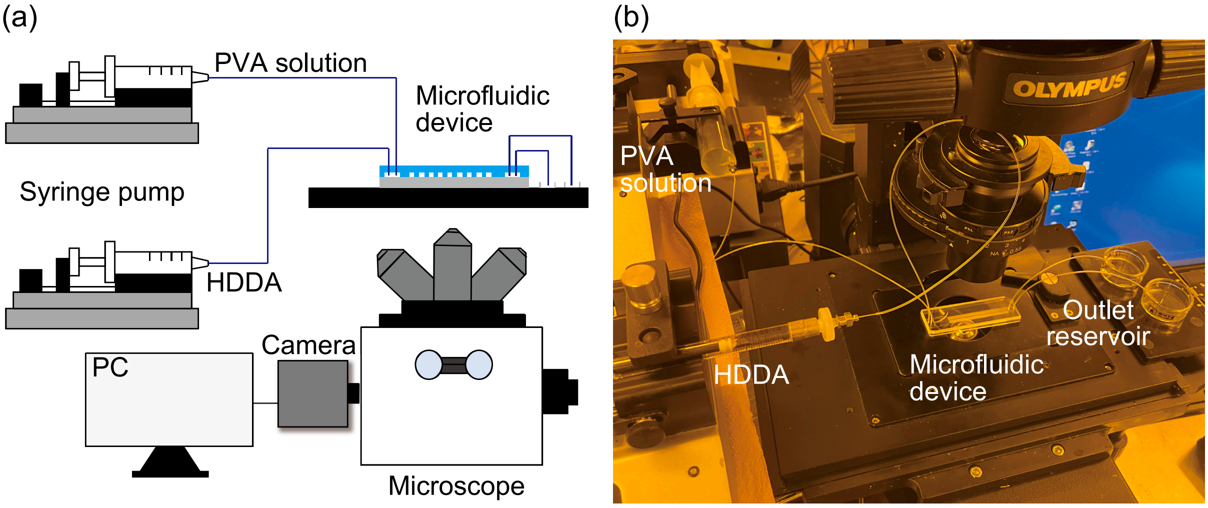

Figure S4. Overall experimental setup. (**a**) A schematic illustration. (**b**) A photograph of the setup.

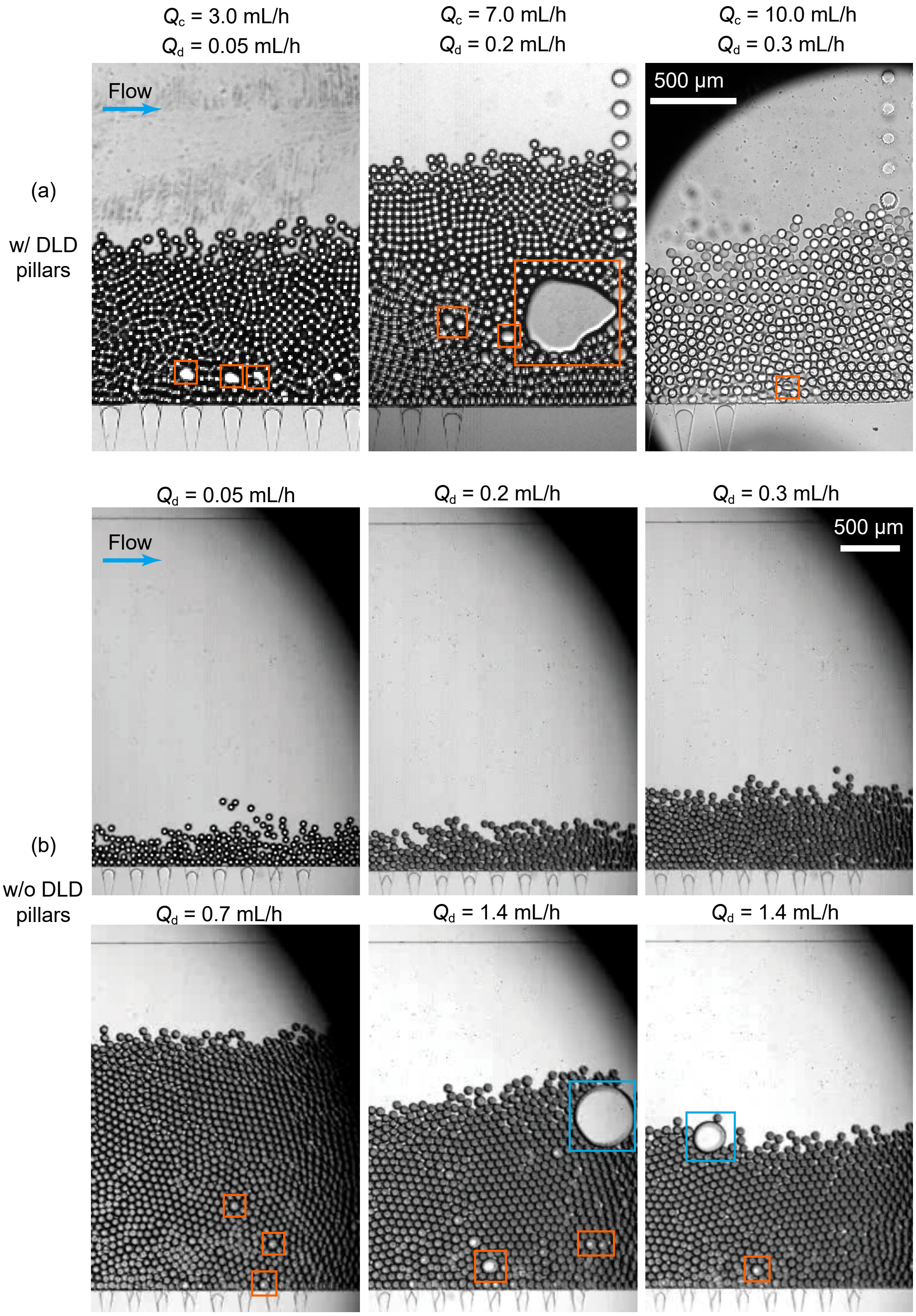

Figure S5. Droplet accumulation and coalescence in the devices (**a**) with and (**b**) without the DLD micropillars at different flow conditions. The orange rectangles denote the coalesced droplets. The light-blue rectangles are the droplets generated in the jetting mode.

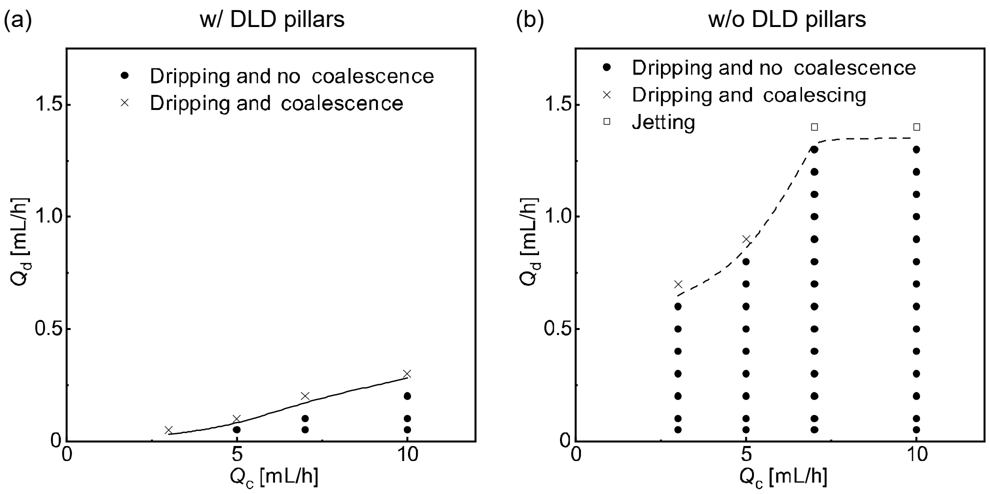

Figure S6. Effect of the flow conditions on the formation of the main droplets and their coalescence. (**a**) A diagram showing the flow condition under which monodisperse main droplets can form and enter the DLD region without coalescence. (**b**) A diagram showing the flow conditions for the step emulsification in dripping and jetting in the no-pillar device.

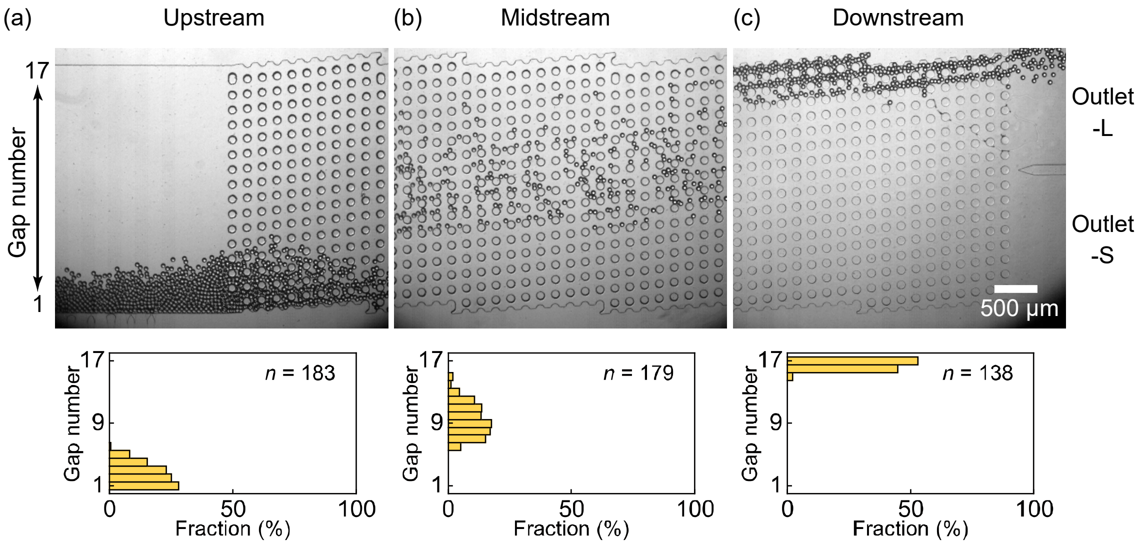

Figure S7. Spatial distribution of main droplets flowing through the DLD pillar arrays when flow rates were Q_d = 0.2 mL/h and Q_c = 10.0 mL/h. (**a**) Main droplets entering the DLD region near the side wall. (**b**) Main droplets flowing through the midstream region in displacement mode. The droplets were counted in the 14th DLD section. (**c**) Fully displaced main droplets entering outlet-L.

.

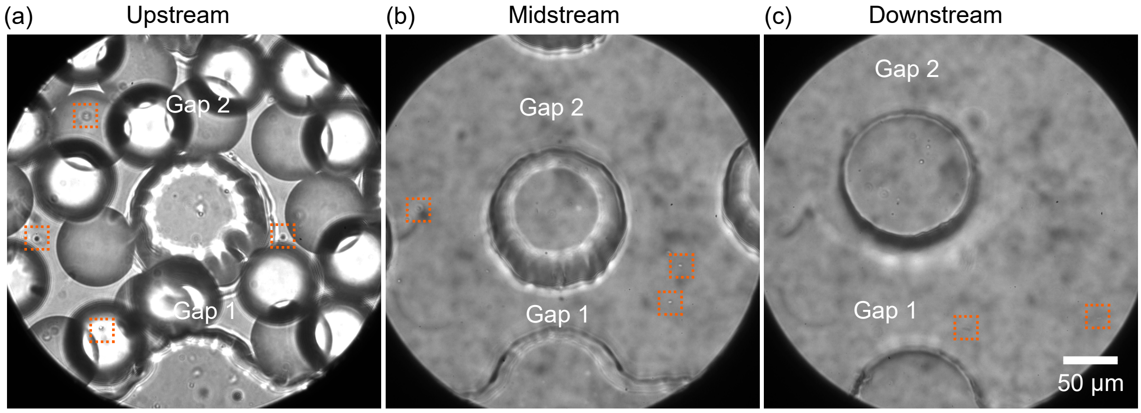

Figure S8. Satellite droplets flowing through the DLD pillars when flow rates were Q_d = 0.2 mL/h and Q_c = 10.0 mL/h. (**a**–**c**) Satellite droplets (in the dashed squares) flowing through the pillars in zigzag mode in the (**a**) upstream, (**b**) midstream, and (**c**) downstream regions.

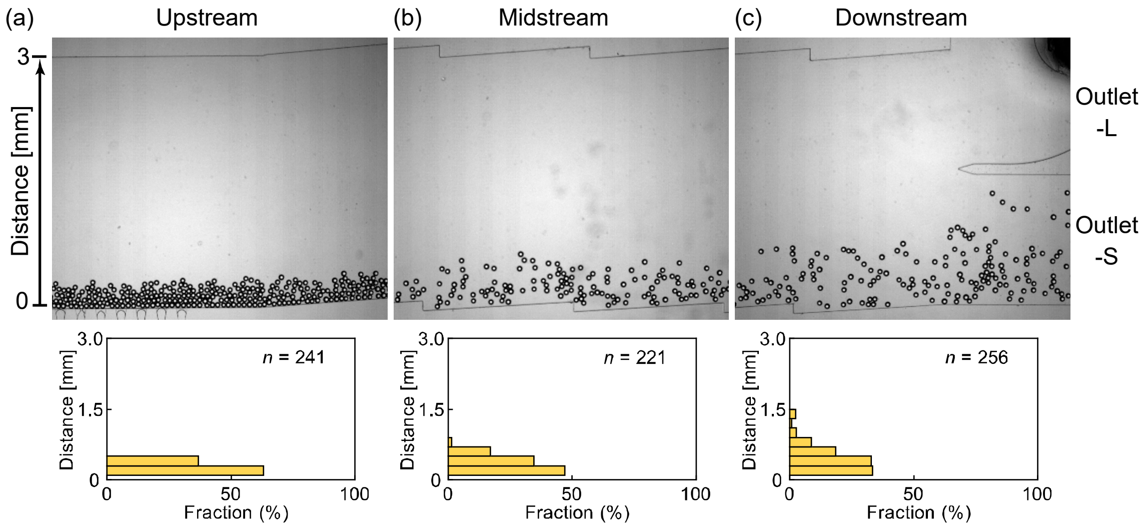

Figure S9. Spatial distribution of main droplets flowing in the no-pillar device when the flow rates were Q_d = 0.2 mL/h and Q_c = 10.0 mL/h. (**a**–**c**) Main droplets flowing through (**a**) the upstream region immediately after the nozzles, (**b**) midstream region, and (**c**) downstream region before the outlets.

Supplementary Movie Captions

Video S1, Movie clip of the step emulsification, recorded at 1000 fps.

Video S2, Movie clip of a magnified view of the break-off of the main and satellite droplets in step emulsification, recorded at 1000 fps.

Video S3, Movie clip of the migration of the main droplets in the (a) upstream, (b) midstream, and (c) downstream regions of the DLD micropillar array, recorded at 500 fps. Scale bar is 3 mm.

Video S4, Movie clip of the migration of satellite droplets in the (a) upstream, (b) midstream, and (c) downstream regions of the DLD micropillar arrays, recorded at 2000 fps. Scale bar is 100 m.

Flow rates of the disperse phase and continuous phase in all videos were 0.1 mL/h and 10.0 mL/h, respectively.

All files are in MEPG-4 format.
